# Beyond Chemotherapies: Recent Strategies in Breast Cancer Treatment

**DOI:** 10.3390/cancers12092634

**Published:** 2020-09-16

**Authors:** Arthur Foulon, Pierrick Theret, Lise Rodat-Despoix, Philippe Kischel

**Affiliations:** 1 Laboratoire de Physiologie Cellulaire et Moléculaire (UR-UPJV 4667), UFR des Sciences, Université de Picardie Jules Verne, 33 Rue St Leu, 80039 Amiens, France; lise.despoix@u-picardie.fr (L.R.-D.); Philippe.Kischel@u-picardie.fr (P.K.); 2 Service de Gynécologie Obstétrique, CHU Amiens Picardie, 80039 Amiens, France; theret.pierrick@chu-amiens.fr; 3 CH Saint Quentin, 1 Avenue Michel de l’Hospital, B.P. 608, 02321 Saint Quentin, France

**Keywords:** breast cancer, metastasis, gene-expression signature, tyrosine kinase inhibitors, CDK4/6 inhibitors, anti-PD-L1

## Abstract

**Simple Summary:**

Breast cancer remains the most frequent women cancer worldwide. The current policy of care for this cancer tends rather at therapeutic de-escalation, with therapies that evolve toward more targeted and more personalized treatments. Personalized medicine requires clinical, but also molecular characterization of tumors, and allows notably chemotherapy to be replaced, or at least used in combination with newer and more appropriate drugs. The aims of this study were (i) to describe recent tools (such as gene-expression signatures) aiding at decision-making in breast cancer management, and (ii) to focus on recent molecules that can be used either in association with chemotherapeutic drugs or after chemotherapies. Such molecules are of utmost importance to help avoid unnecessary chemotherapies. When substitution treatments are available (in early breast cancer for instance), a big step can be made toward personalized medicine for the patient’s benefit. This clinical strategy is a medical challenge for the upcoming years.

**Abstract:**

In 2018, about 2.1 million women have been diagnosed with breast cancer worldwide. Treatments include—among others—surgery, chemotherapy, radiotherapy, or endocrine therapy. The current policy of care tends rather at therapeutic de-escalation, and systemic treatment such as chemotherapies alone are not systematically considered as the best option anymore. With recent advances in the understanding of cancer biology, and as a complement to anatomic staging, some biological factors (assessed notably via gene-expression signatures) are taken into account to evaluate the benefit of a chemotherapy regimen. The first aim of this review will be to summarize when chemotherapies can be avoided or used only combined with other treatments. The second aim will focus on molecules that can be used instead of chemotherapeutic drugs or used in combination with chemotherapeutic drugs to improve treatment outcomes. These therapeutic molecules have emerged from the collaboration between fundamental and clinical research, and include molecules, such as tyrosine kinase inhibitors, CDK4/6 inhibitors, and monoclonal antibodies (such as anti-PD-L1). In the fight against cancer, new tools aiding decision making are of the utmost importance: gene-expression signatures have proven to be valuable in the clinic, notably, to know when chemotherapies can be avoided. When substitution treatments are also available, a big step can be made toward personalized medicine for the patient’s benefit.

## 1. Introduction

As the first woman-related cancer in the US [1] and worldwide [2], breast cancer (BC) remains a complex public health issue. Treatments include surgery, chemotherapy, radiotherapy, or endocrine therapy, among others. The current policy of care tends at therapeutic de-escalation and toward more personalized treatments (depending, among others, on histological classifications, Scarff–Bloom–Richardson (SBR) grade, hormone receptor (HR) status, human epidermal growth factor receptor 2 (HER2) expression, and Ki67 index). Despite these adjusted treatments, local or distant recurrence inexorably still occurs, leading patients to die [3].

Therefore, management of patients requires constant improvements, and efficient therapeutic molecules are still needed. Chemotherapies are not always the best option, as their induced toxicities remain a major concern. At the dawn of the 2020s, new tools are available for decision making. Gene-Expression Signature (GES) for instance can now be used to decipher the heterogeneity of BC, and are used in many countries for chemotherapy decisions in estrogen-receptor-positive (ER^+^), HER2-negative (HER2^−^) early BC [4]. Our aim is first to summarize when chemotherapies can be avoided or used only combined with other treatments, using notably GES. When no benefits are expected from chemotherapy regimens, new solutions have to be found to improve patient outcomes. We now have gained insight into pros and cons of several molecules that have been introduced in the last two decades. The aim of this review is, therefore, to also highlight three of the most helpful and/or promising recent molecules in BC treatments to date (one for each main BC subtype: one for HER2^+^ patients, one for triple-negative breast cancer (TNBC) patients, and one for HR^+^/HER2^−^ patients, with neratinib, atezolizumab, and palbociclib, respectively). For each molecule, we will detail effects on disease-free survival (DFS, survival time without recurrence of the disease), overall survival (OS, survival time before death), and progression-free survival (PFS, time before progression of the disease, primarily in metastatic situation).

## 2. Management of Breast Cancer: When Chemotherapy Should Be Avoided?

**Figure 1:**
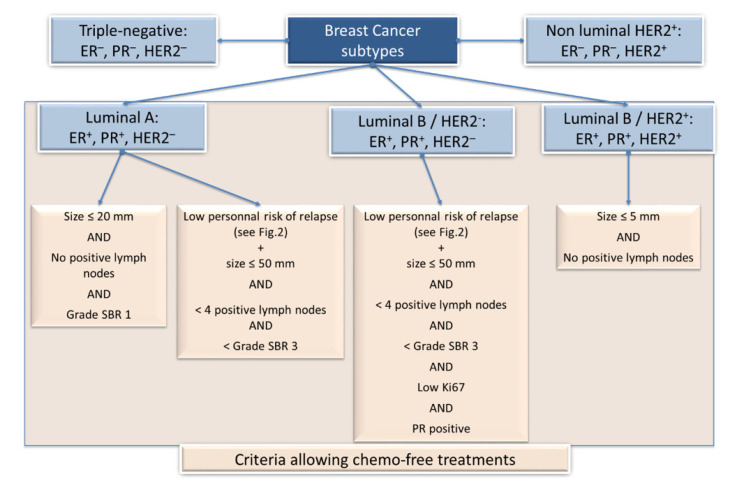
Breast cancer histological subtypes (**blue** color) and clinical criteria allowing to avoid chemotherapy (**orange** color). Among the five intrinsic subtypes (that either luminal, non-luminal, or triple-negative), three of them (the luminal ones) can be associated to chemo-free treatments following specific clinical criteria (tumor size, lymph nodes, Scarff–Bloom–Richardson (SBR) grade, Ki67, and progesterone receptor (PR) status).

Nowadays, clinical practice typically uses a classification of five subtypes on the basis of histological and molecular characteristics, such as (among others) estrogen receptor (ER), progesterone receptor (PR, tumors ER^+^ and/or PR^+^ being considered hormone receptor (HR) positive, i.e., HR^+^), HER2, and Ki67 expression (see [4] for review and Figure 1):

The Luminal A subtype is typically ER^+^/PR^+^/HER2^−^/low Ki67 index/low grade and is of good prognosis.

The Luminal B HER2^−^ subtype is ER^+^/PR^+^/HER2^−^/high Ki67 index/higher grade and is of intermediate prognosis.

Luminal B HER2^+^ subtype is ER^+^/PR^+^/HER2^+^/high Ki67 index and is also of intermediate prognosis.

The non-luminal HER2^+^-enriched subtype is ER^−^/PR^−^/HER2^+^/high Ki67 index and is still of intermediate prognosis.

The triple-negative breast cancer (TNBC) subtype does not express ER, PR, or HER2, has high Ki67 index, and is of poor prognosis.

Following histological classifications, SBR grade, tumor characteristics (size, axillar node involvement), and molecular classification, therapy will be adapted, depending on the presence or absence of metastases.

In early BC (without detectable metastases), women will undergo surgery if considered as operable. Most of these patients will, however, need additional systemic therapies: (i) before surgery (neoadjuvant therapy), when reducing the tumor burden is desired (inflammatory tumors, big-sized TNBC tumors, big lesion in small breast in order to avoid mastectom), or when pathologic complete response (pCR, i.e., lack of all signs of cancer in tissue samples after treatment) is strongly correlated with outcome, such as in TNBC and HR^−^/HER2^+^ treated with trastuzumab [5] or (ii) after surgery (adjuvant therapy).

Chemotherapies have proven to be successful treatments. However, chemotherapies may be administered unnecessarily, and sparing chemotherapy-associated morbidity is, therefore, absolutely essential, provided adequate tools are available. From the five surrogate intrinsic subtypes (represented in Figure 1), three can be concerned by chemotherapy-free treatments (luminal A and B subtypes), provided they meet specific requirements. The Figure 1 summarizes the criteria to be fulfilled for skipping chemotherapy that would not bring any improvement in the outcome.

For neoadjuvant therapy, Connolly and collaborators have shown that PET scans could identify BC patients who may receive HER2-directed therapy alone and avoid chemotherapy [6]. Pertuzumab and Trastuzumab are used in this neoadjuvant therapy in women with stage II/III ER^−^/HER2^+^ BC.

**Figure 2:**
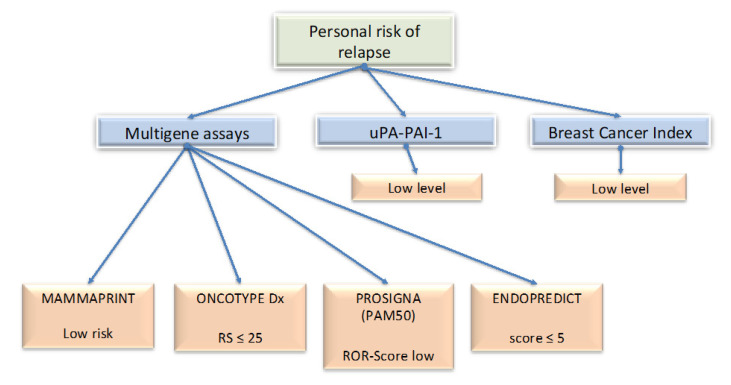
Contribution of the gene-expression signature (GES) in the chemo-free treatments decision in estrogen-receptor-positive (ER^+^) human epidermal growth factor receptor 2-negative (HER2^−^) breast cancer. Here are summarized the most common genetic tests used, allowing to discriminate patients for whom chemotherapy could be avoided.

The question as to whether ER^+^/HER2^−^ early BC patients need chemotherapy (neoadjuvant or adjuvant) in addition to endocrine therapy is highly relevant. In patients with luminal A disease and with low tumor burden, chemotherapy should be omitted. In general, the recommendation for chemotherapy in ER^+^, HER2^−^ tumors may be influenced by proliferation (Ki67 expression) and possibly the results of a GES (Figure 1 and Figure 2). Criteria to avoid chemotherapy within patients with Luminal B disease are more stringent. Notably, patients with Luminal B must have a low personal risk of relapse (determined by GES) to avoid chemotherapy.

In invasive BC, adjuvant therapy indications depend on clinical anatomo-pathological criteria, but for these advanced BCs, skipping chemotherapy is much more challenging. However, this therapy does not remain necessarily indicated for some patients [7]. Indeed, chemotherapy would not bring benefits to luminal-like metastatic HER2^−^ BC with axillar node invasion (N^+^) with strong HR expression [8]. Instead, endocrine-based therapies are used until endocrine resistance occurs, unless progression is rapid or severe organ dysfunction appears [4,9]. A CDK4/6 inhibitor (such as palbociclib, see paragraph 4.3 below) can be used concomitantly [9].

## 3. Management of Breast Cancer: New Tools for Decision Making

Many adverse events can occur after chemotherapy, some of them are harmful and can even be fatal: it is thus necessary to develop some tests (GES for instance) or identify tumor markers (uPA-PAI-1, for example) to improve and refine the selection of patients for whom chemotherapy could be avoided.

GES can help determine which patients need adjuvant chemotherapy. Four tests are currently available: two first-generation GES (OncotypeDX from Genomic Health Inc., Redwood City, CA, USA and MammaPrint from Agendia BV, Amsterdam, The Netherlands), performed directly by the company itself, and two second-generation GES (Endopredict from Myriad Genetics Inc, Salt Lake City, UT and Prosigna from NanoString Technologies, Seattle, WA, USA) that can be obtained on dedicated devices.

### 3.1. OncotypeDX

This test use reverse transcriptase-quantitative polymerase chain reaction (RT-qPCR) to assess the expression of 21 genes (listed in Table S1). Among them, 16 genes are linked to proliferation, HER2, and HR expressions. The result is given as recurrence score (RS), ranging from 0 to 100: low risk 0–10, intermediate 11–25, and high risk 26–100 [10], correlated with the distant recurrence risk at 10 years. In a TailoRx study, 10,253 patients with HR^+^, HER2^−^ and axillary node-negative BC were included. 1616 patients had low risk recurrence score and received endocrine therapy alone with no chemotherapy. The five-year invasive DFS was 93.8% and five-year overall survival rate was 98% [11]. To precise the role of OncotypeDX in patients with HR^+^, HER2^−^, and one to three lymph nodes involved, the RxPONDER study was designed: results are expected in two years [12].

Two other studies assessed clinical validation of OncotypeDX [13,14]. In both studies, thousands of patients with HR^+^, HER2^−^, and axillary node-negative BC were included. This test perfectly selects patients at low risk of recurrence for whom chemotherapy would not be a good option. The American Society of Clinical Oncology (ASCO) recommend that women with HR^+^/HER2^−^ early BC older than 50 may skip chemotherapy if their Oncotype DX recurrence score (RS) is in the intermediate range (11–25) or lower.

### 3.2. MammaPrint

Known as the “70-gene signature” (list in Table S1), this test gives risk of distant recurrence at five or 10 years. In the MINDACT study, Cardoso and coworkers enrolled 6693 patients with BC classified in four groups [15], with either low or high clinical risk combined with either low or high genomic risk. In patients with high clinical risk and low genomic risk, adjuvant chemotherapy was not associated with higher distant metastasis free survival (DMFS): for these patients, the absence of chemotherapeutic treatment on the basis of the 70-gene signature led to a five-year rate of survival without distant metastasis that was 1.5 percentage points lower than the rate with chemotherapy. Same results were found in patients with low clinical risk and high genomic risk: adjuvant chemotherapy was not associated with higher DMFS. Given these findings, approximately 46% of women with breast cancer who are at high clinical risk might not require chemotherapy [15]. Patients included in this study had T1, T2 or T3 BC, N0, N1 or N2 BC, ER^+^ or ER^−^, and HER2^+^ or HER2^−^ BC tumors. BC characteristics were used to define the clinical risk group.

Both MammaPrint and OncotypeDX are used to select ER^+^/HER2^−^ patients for whom chemotherapy could be avoided [14].

### 3.3. Endopredict

This test gives a score (ranging from 0 to 15) in order to predict distant recurrence at five or 10 years. Expression of 11 genes is tested by RT-qPCR (list in Table S1). In 2011, Filipits and coworkers assessed Endopredict in 1330 patients with BC [16]. The threshold of Endopredict score to distinguish low vs. high-risk was 5. Fitzal and collaborators have assessed local recurrence-free survival (LRFS) in 1324 patients with BC [17]: LRFS was significantly higher in patients with low Endopredict score. Predictions of metastasis have also been assessed in 1702 patients [18]. Absence of distant recurrence rate was significantly higher in low-risk group. 

### 3.4. Prosigna (PAM50)

This test analyzes 50 genes expression by RT-qPCR (list in Table S1). It gives a score (ranging from 0 to 100) by an algorithm that takes into account tumor size (≤2 cm or >2 cm) and node positive status (0 to 3). The algorithm outputs a “risk of recurrence” score (ROR-score), correlated with recurrence probability at ten years. PAM50 ROR score and ROR-based risk groups can differentiate BC patients with respect to their risk for late distant recurrence beyond what could be achieved with classical clinicopathologic risk factors [19]. In another study including 1478 patients, ROR score was associated with distant recurrence free-survival (DRFS) rates [20]. A 10-year metastasis risk of < 3.5% in the ROR low category makes it unlikely that additional chemotherapy would improve this outcome, not to mention the unfavorable harm/benefit ratio with respect to treatment side-effects. PAM50, thus, help to avoid unwarranted overtreatment [20].

Both Prosigna and Endopredict tests also select ER^+^/HER2^−^ patients with low local or distant risk of recurrence for whom chemotherapy could be avoided [21].

### 3.5. uPA-PAI1

Antigen content of Fibrinolytic factors urokinase-type plasminogen activator (uPA) and its inhibitor type 1 (PAI-1) correlate with BC aggressiveness. Indeed, in BC, a high level of uPA-PAI1 is correlated with higher risk of recurrence and poorer overall survival [22]. Patients with node negative and low uPA and PAI-1 have an estimated five-year overall survival, going up to 95% without any adjuvant therapy (low level was defined as uPA ≤ 3 ng/mg and PAI-1 ≤ 14 ng/mg) [23].

### 3.6. Breast Cancer Index (BCI)

The Breast Cancer Index (BCI) was developed using an ER^+^ early stage BC cohort [24]. This test is based on the ratio between the two genes, *HOXB13* and *IL17BR* (H/I), and on the molecular grade index (MGI). The combination of H/I and MGI generates a prognostic score quantifying overall distant recurrence risk (0–10 years), but also late (5–10 years) distant recurrence risk. H/I cut off is 0.06 to distinguish low from high-risk group. BCI can predict risk of late recurrence for patients with ER^+^ BC after five years of tamoxifen [25], but also early recurrence risk [26].

### 3.7. Limitations

At this time, these tests remain unfortunately restricted to a limited population of patients. The main problems in current practice are accessibility and cost of these tests. Depending on these two criteria, some patients for which these test would be clearly indicated will not be able to benefit from these tests because of availability or reimbursement problems. It is, therefore, essential to find ways to generalize the use and the accessibility of these tests that have shown reliability in terms of prognostic utility. Further studies, with even longer follow-up, would allow to more precisely define this prognostic value, but also to better select patients.

Prognostic value of MammaPrint and OncotypeDx are of level of evidence IA: this is not the case for the two other tests. More studies about economic and clinical utility for PAM50 and Endopredict are urgently needed.

## 4. Promising Molecules of the 2010’s Decade

Since chemotherapeutic drugs alone are not always the best option for BC treatment, alternatives have to be found. Therapeutic molecules have emerged from the collaboration between fundamental and clinical research and include molecules, such as tyrosine kinase inhibitors, CDK4/6 inhibitors, and humanized monoclonal antibodies, among others. For each main BC subtype (HER2^+^, TNBC, and HR^+^/HER2^−^), a promising molecule (that can be used either alone or in combination) will be described.

### 4.1. Neratinib (for HER2^+^ Patients)

HER2 overexpression status represents 15% of all BC [27]. Those BC are more aggressive, with a poorer prognosis than those without HER2 overexpression [28]. *HER2* gene amplification involves increased cell proliferation, local recurrence, and metastatic progression [29]. Trastuzumab has been the standard of care for the last 20 years for HER2 positive early and advanced BC. Trastuzumab therapy without chemotherapy in early BC has been repeatedly discussed in international consensus meetings but is compromised by the lack of solid evidence from clinical studies. Therefore, trastuzumab plus chemotherapy remains the preferred option in all patients with HER2^+^ early BC for which adjuvant treatment is indicated [30]. Despite this specific treatment, 15 to 25% of patients will have recurrence or metastatic progression [31,32], due to—among other things—acquired resistance [33,34]. Furthermore, the risk of relapse is higher during the first 12 months after trastuzumab therapy [35]. For one decade, new molecules have been found for HER2 overexpressing BC. Among them, Neratinib is an irreversible tyrosine kinase inhibitor (TKI) of HER1, HER2, and HER4.

#### 4.1.1. Primary Molecular Function and In Vitro and In Vivo Assessments

HER2 is a member of the epidermal growth factor receptor (EGFR) family. This family is composed of four proteins: HER1 (EGFR), HER2, HER3, and HER4. Many ligands interact with the HER family members (excluding HER2) to control cell proliferation, survival, differentiation, and motility [36]. Each HER family member contains a fully functional intracellular tyrosine kinase (TK) domain, with the exception of HER3.

Neratinib interacts with the catalytic domain of HER1, HER2, and HER4 [37]. In a cell-free autophosphorylation assay, Neratinib was able to reduce HER2 and EGFR activities by ca. 50% at concentrations of 59 nM and 92 nM, respectively [38]. Decrease in phospho-HER2 was found to be dose- and time-dependent [39]. To reduce 50% of the activity of other TK proteins, such as Akt or cyclin D1/CDK4, concentrations have to be raised at more than 20 µM, making Neratinib a potential selective inhibitor of HER2 [38]. In vitro, Neratinib inhibits cell proliferation of HER2-overexpressing cell lines but has only minor effects on HER2 negative cell lines [38].

Interestingly, Neratinib was able to decrease ligand-independent receptor phosphorylation and EGF-dependent phosphorylation of EGFR in BT474 cells and A431 cells, respectively [38]. This prevents the Akt-mediated signal transduction and activation of MAPK pathways. Neratinib also modulates cell-cycle progression by reducing cyclin D1 expression and the phosphorylation of the Retinoblastoma (Rb)-susceptibility gene [38]. Simultaneously, expression of p27 (cell-cycle progression inhibitor) was found to be increased. Last but not least, in vivo data showed dose-dependent tumor growth reduction by Neratinib in female athymic nude mice, associated with a HER2 phosphorylation decrease (by 84%) 1h after oral dose of 40 mg/kg of Neratinib [38].

#### 4.1.2. Clinical Evidences

Two phase III clinical trials (named ExteNET trials) investigating benefits of Neratinib treatments have been recently published by Chan and collaborators [40] and Martin M. and coworkers [41]. The study from Martin M et al. is actually an extension from Chan et al. with five years follow-up data about DFS. The ExteNET trial is a multicentered, randomized, double-blinded, placebo-controlled phase 3 trial: 2840 patients were included to receive neratinib or placebo (1420 in each group). Patients had confirmed HER2 overexpression and had received at least one year of trastuzumab therapy, without local recurrence or metastasis. Primary endpoints were events defined as invasive local or distant recurrence or death from any cause. Neratinib significantly reduced the number of recurrence at two and five years after inclusion. Neratinib was more efficient in HR^+^ patients and plays an obvious role in adjuvant situation. Phase III studies showed improvement in DFS for all patients with HER2-overexpressed BC.

Regarding the phase II trials, six studies focusing on neratinib’s efficiency, either in monotherapy or associated with paclitaxel or trastuzumab, in patients with advanced or metastatic HER2^+^ BC (FIGO IIIB, IIIC or IV), retained our attention. Other associations were excluded to avoid confusion with multi-chemotherapies regimens. From these six studies, 662 patients were included to receive neratinib monotherapy or in association with paclitaxel or trastuzumab. For those patients, neratinib had encouraging positive antitumor activity either in monotherapy [42,43,44] or in association with paclitaxel [45,46] or trastuzumab [47]. Moreover, efficiency of neratinib was not inferior than other reference treatments for advanced or metastatic HER2^+^ BC (trastuzumab plus paclitaxel [45] or lapatinib plus capecitabine) [44]. Neratinib could therefore be used for patients with resistance or major adverse events to reference treatments. All data from phase II studies are summarized in Table S2.

In view of these results, neratinib plays a role in adjuvant and metastatic treatments, with phase III studies showing improvement in DFS for all BC patients overexpressing HER2. This was more pronounced for patients with HR expression [41].

#### 4.1.3. Neratinib and Main Adverse Events

In all studies, grade 3 or 4 diarrhea were the primary adverse events; 38.9% (865/2222) of patients unfortunately suffered from these symptoms.

#### 4.1.4. Other Associations with Trastuzumab and Potential Limitations

The association pertuzumab (which inhibits HER2 heterodimerization) with trastuzumab is also promising. 4805 patients with early HER2^+^ BC have been included after adjuvant chemotherapy to receive either one year trastuzumab + placebo or one year trastuzumab + pertuzumab. The three-year invasive DFS was significantly improved in the pertuzumab + trastuzumab group [48]. The most frequent adverse event for the pertuzumab group was grade 3 neutropenia (16.3%) and 12.1% febrile neutropenia, but only 9.8% grade 3 diarrhea [48]. When docetaxel is added to trastuzumab + pertuzumab, significantly higher rates of complete response in neoadjuvant settings were obtained when compared to trastuzumab + docetaxel [49].

TKI have also been tested in association with trastuzumab. In neoadjuvant settings, the association between lapatinib + trastuzumab allows a higher complete response rate when compared to lapatinib or trastuzumab alone [50]. Studies of anti-HER2 molecules in neoadjuvant settings have shown a correlation between complete response and DFS [51]. This association also permits improvements in PFS in metastatic HER2^+^ BC patients [52]. More recently, the association between trastuzumab + capecitabine + tucatinib has been shown to allow significant improvement of PFS in HER2^+^ metastatic BC patients [53].

The use of neratinib in early HER2^+^ BC treatment after one year of trastuzumab allows an increase in DFS. Its use in a metastatic setting has also shown encouraging results. However, these results are somewhat “tarnished” because of the main side effect of neratinib. Indeed, digestive disorders (mainly grade 3 or 4 diarrhea) are frequently found with this molecule. Patients with early HER2^+^ BC tumors > 2 cm are now encouraged to accept neoadjuvant chemotherapy [21]. The association between pertuzumab + trastuzumab + docetaxel in this setting is also promising.

The contribution of neratinib remains to be studied and defined after surgery for these patients that have already received two anti-HER2 treatments in neoadjuvant settings, and also in association with trastuzumab in neoadjuvant settings (similarly to the association pertuzumab/trastuzumab +/− docetaxel), preferably with optimal prevention of digestive side effects.

### 4.2. Atezolizumab (for TNBC Patients)

#### 4.2.1. Primary Molecular Function and In Vitro and In Vivo Assessments

Breast cancers with no estrogen nor progesterone-receptors expression and without HER2 overexpression are named triple-negative BC (TNBC). About 10% of BC are TNBC [54]. Those cancers are characterized by a poorer prognosis for the patients: higher distant recurrence, higher death rate, and higher metastatic spread [54,55,56]. In a non-metastatic situation, available treatments include surgery, radiotherapy, and adjuvant chemotherapy. Concerning metastatic disease in TNBC, chemotherapy is the only key for treatment. Numerous molecules are used: taxol, carboplatin, capecitabin, etc. Chemotherapeutic drugs can be switched when disease evolves or when severe side-effects occur. Hence, novel therapeutics that could prolong life expectancy when associated with chemotherapeutic drugs (or even used alone) are, therefore, highly valuable for TNBC.

In 2012, Liu et al. found that higher rates of intratumoral CD8^+^ T cell infiltration could be considered as an independent good prognostic factor [57]. In addition, in TNBC, tumor-infiltrating lymphocytes (TIL) are of prognostic value: the more there are, the better the survival and the lower the risk of recurrence [58,59]. TIL include CD8^+^ lymphocytes, CD4^+^ T helpers, natural killers, and B-cells [60]. These elements strongly suggest a role for immunotherapy in TNBC. PD-1 (programmed cell death protein-1) is an immune checkpoint, expressed on activated T-cells, that downregulate T cells activity when it binds to one of its ligand, namely PD-L1. When PD-L1 is bound to the 40 kDa transmembrane protein PD-1, cancer cells are able to escape the immune response and, therefore, cell death [61]. PD-L1 expression increases invasiveness and tumorigenesis of cancer cells by reducing their sensitivity to T-cells mediating lysis [62]. In BC, PD-L1 expression is associated with high histologic grade, HR^−^, HER2^−^ and elevated Ki67 [63,64].

PD-L1 was, therefore, proposed in the early 2000s as a potential target in cancer immunotherapy in human clinic [65]. Interestingly, it is also associated with the expression of the tumor suppressor gene PTEN (phosphatase and tensin homolog). PTEN loss occurs in 30% of BC patients [66]. In glioblastoma, deletion or silencing of PTEN led to increased expression of PD-L1 [67]. Mittendorf and collaborators found that PTEN downregulation led to increased PD-L1 expression: this overexpression was able to cause decreased proliferation and increased apoptosis of T-cells, playing a major role in the antitumor immune response [68]. Recently, Barroso-Sousa et al. established that patients with TNBC treated with anti-PD-L1 therapies and presenting PTEN alterations were associated with shorter survival [69].

Atezolizumab is a humanized monoclonal antibody produced in Chinese hamster ovary cells and developed by Genentech, which is able to bind PD-L1. The link between PD-L1 and atezolizumab occurs around the upper side, close to the N-terminus of PD-L1 [70]. It inhibits binding of PD-L1 to receptor PD-1. The interaction of atezolizumab with PD-L1 is stronger than PD-L1/PD1 interaction [70]. In vitro, atezolizumab potentiate T-cell-mediated toxicity by increasing cell apoptosis in a dose-dependent manner only in cell lines overexpressing PD-L1 [71]. Atezolizumab also decreased FAK phosphorylation, inhibiting cell invasion and motility [71].

#### 4.2.2. Clinical Evidences

Atezolizumab efficiency in locally advanced or metastatic TNBC was assessed in one phase III study [72] and two phase 1b studies [73,74]. In the phase III study, 902 patients received nab-paclitaxel, either in association with atezolizumab (*n* = 451) or with placebo (nab-paclitaxel monotherapy, *n* = 451). Atezolizumab plus nab-paclitaxel prolonged PFS among patients with metastatic TNBC in both the intention-to-treat population and the PD-L1–positive subgroup. PD-L1 expression was found in 185 patients (41%) from the atezolizumab group and in 184 patients (40.8%) from the placebo group.

The study of Schmid and collaborators was updated in 2020 [75]. In the PD-L1 immune cell-positive population, the updated median PFS was 7.5 months with atezolizumab and 5.3 months with placebo [75]. Regarding the OS analysis in patients with PD-L1 immune cell-positive tumors, treatment with atezolizumab increased the median OS to 25 months versus 18 months in the placebo group.

The association [atezolizumab + nab-paclitaxel] was also assessed in a phase 1b study and showed promising effects. Thirty-three patients with advanced or metastatic TNBC, without more than two chemotherapy regimens before, were included [73]. Another phase 1b study assessed efficiency of atezolizumab in monotherapy on 116 patients with metastatic TNBC [74]. Data are summarized in Table S3.

#### 4.2.3. Atezolizumab and Main Adverse Events

In the study of Schmid and collaborators, peripheral neuropathy and neutropenia were described as the main grade 3 or 4 adverse events: 5.5% had peripheral neuropathy (25/452) and 8.2% (37/452) had neutropenia [72]. In the study of Adams and collaborators, grade 3 or 4 neutropenia or peripheral neuropathy were reported on 45.5% (15/33) and 3% (1/33) of patients, respectively [73]. These adverse events could be attributed to paclitaxel.

#### 4.2.4. Limitations

Main results concerning atezolizumab are based on one main phase III study. At this time, current results only concern the use of this molecule in the metastatic setting. Gains in terms of survival, although significant, can appear relatively weak but are nonetheless substantial for these TNBC patients. Numerous studies concerning adjuvant treatment of early stage TNBC patients are ongoing (NCT03498716, NCT01898117, NCT03802604, for instance), and results are highly awaited. These results will preferably have to be validated in adjuvant settings, proving, therefore, that this molecule improve DFS of TNBC patients. Similarly, the use of atezolizumab in neoadjuvant settings could perhaps allow an increase in complete response rate and, thus, increase DFS. Numerous studies are ongoing to address these two questions.

### 4.3. Palbociclib (for HR^+^/HER2^−^ Patients)

Around 80% of BC express hormone receptors (estrogen and progesterone). Less than 10% of these ER^+^ or PR^+^ patients overexpress HER2. Endocrine therapy reduces time to relapse for those patients with HR^+^ cancers. Endocrine therapy is also the reference for non-symptomatic metastatic situation [76]. Despite this treatment, many patients will experience relapse during or after adjuvant endocrine therapy.

Palbociclib is a selective, potent, and orally available inhibitor of cyclin-dependent kinases 4 and 6 (CDK4/6). It has been shown to be preferentially active in luminal preclinical models of BC [77,78], and to be synergistic with endocrine therapy [79,80]. Palbociclib is approved in the first line for HR^+^ HER2^−^ advanced BC.

#### 4.3.1. Primary Molecular Function and In Vitro Assessment

It has been established that dysregulation of [cyclin D1:CDK4/6] complex is one of the events inducing BC initiation and progression [81]. Moreover, cyclin D1 and CDK4 are key factors for BC induction in mouse models [82]. Retinoblastoma gene product (pRb) is able to hold captive transcription factors from E2F family, leading to cell cycle progression blockade. Stimulated by some mitogenic or adhesion signals, quiescent cells produce cyclin D1. This latter will form complexes with CDK4/6, initiating phosphorylation of pRb. This phosphorylation releases E2F, allowing transcription of S-phase target genes and then proliferation [81]. CDK4 and cyclin D1 amplification occur in 15–25% of BC [83]. Signaling from estrogen receptors (ER) leads to increased cyclin D1 expression that up-regulate CDK4/6 activity [84]. This latter mechanism can be estrogen-independent, explaining resistance to classic endocrine therapy. Moreover, cyclin D1 can independently activate ER [85]. Endocrine therapies have different effects on ER pathways: for instance, tamoxifen is an ER antagonist, aromatase inhibitors block estrogen production, and fulvestrant down-regulates ER expression. Palbociclib, an anti-CDK4/6 inhibitor, strongly down regulates cancer cells proliferation with dephosphorylation of pRb and a decrease in E2F-dependent gene expression [86]. This orally small molecule has a high level of selectivity for CDK4 and CDK6.

#### 4.3.2. Clinical Evidence

Preclinical studies of palbociclib have shown its ability to preferentially inhibit the growth of ER^+^ BC cells, act synergistically with anti-estrogens, and reverse endocrine resistance. These findings led to the design and implementation of PALOMA (Palbociclib: Ongoing Trials in the Management of BC)–1, a randomized, study designed to evaluate [palbociclib + letrozole] vs. letrozole alone as first-line therapy in post-menopausal women with ER^+^/HER2^−^ advanced BC [87,88]. In this proof-of-concept study, median PFS was significantly higher in the palbociclib arm.

We selected three phase II studies assessing palbociclib [87,89,90]. 243 patients were enrolled to receive either palbociclib alone (33 patients) [90] or in association with letrozole (210 patients [89]). Median PFS ranged from 3.8 to 20.2 months, with a median follow-up ranging from 14.2 to 29.6 months (Table S3).

Palbociclib has been assessed in two phase III studies: PALOMA-2 (assessing palbociclib + letrozole vs. placebo + letrozole) and PALOMA-3 (assessing efficiency of palbociclib + fulvestrant vs. placebo + fulvestrant) in women with HR^+^/HER2^−^ metastatic BC who progressed after endocrine therapy [87,91]. Median PFS ranged from 9.5 to 24.8 months for the 791 patients enrolled in palbociclib arms (444 with letrozole and 347 with fulvestrant) and from 4.6 to 14.5 months for the placebo arms. Differences were significant for palbociclib group: HR was 0.46 with fulvestrant and 0.58 with letrozole. OS data are not available for each study.

Median PFS was higher when palbociclib was assessed in association with letrozole compared to palbociclib + fulvestrant (PALOMA 1, 2, and 3). The worst results in terms of median PFS and progression rate were from the DeMichele study, where palbociclib was assessed in monotherapy [90]. Data are summarized in Table S4.

#### 4.3.3. Palbociclib and Main Adverse Events

Neutropenia is the primary grade 3 or 4 adverse event induced by palbociclib: in PALOMA-2 and PALOMA-3 for instance, 438 (55.3%) and 80 (10.1%) patients experienced grade 3 and 4 neutropenia, respectively [91]. However, three cases of febrile neutropenia have been found in the palbociclib group vs. only one in the placebo group [91]. Other TKIs have also been reported to induce febrile neutropenia (ribociclib + letrozole: 2% [92], abemaciclib + fulvestrant: <1% [93]). When compared to others chemotherapy protocols for BC, febrile neutropenia occurs rarely (1%) when paclitaxel is weekly delivered [94], much more frequently (around 10%) with anthracyclins and docetaxel/cyclophosphamide [95], and more frequently (above 20%) with fluorouracil/epirubicin/cyclophosphamide [96].

#### 4.3.4. Limitations

These results obtained with palbociclib are certainly promising but come from only two phase III studies, enrolling less than 1000 patients (791 exactly). Furthermore, palbociclib has been associated to two different molecules: letrozole [87] and fulvestrant [91]. Moreover, some patients enrolled in PALOMA-3 study were treated with first line chemotherapy before inclusion, and this contrasts with the PALOMA-2 study. More phase III studies, including larger cohorts with patients without first-line chemotherapy, should be performed for each association. Its role in adjuvant or neoadjuvant situation for early stage HR^+^/HER2^−^ BC remains also to be defined: many studies are in progress on this matter.

## 5. Discussion

Breast cancer is a complex disease, and there are potentially as many treatments as cancer subtypes. To distinguish these subsets, clinicians pay attention to patient’s age, tumor size, nodal status, metastatic status, and pathological data, such as ductal or lobular subtypes, histological grades, HR status, HER2, and Ki67 expression. In this way, breast cancer is already the pioneer of personalized medicine, but going ahead and bringing personalization a step further remains always possible and is of the utmost importance. Indeed, although efficiency of chemotherapy has been proved for decades for patients with BC in both neo-adjuvant and adjuvant regimens, and although radiotherapy, endocrine and anti-HER2 therapies are also largely used with success, the only important thing to consider is that patients still die from BC.

Knowledges about the tumor characteristics combined with personalized treatments are the best option to reduce mortality. Chemotherapy for instance is far from being a trivial treatment: chemotherapy-induced toxicity remains a major concern (adverse events can be serious and even lethal), leading to the question as to whether other monotherapies (or at least associations with other molecules) could be adequate alternative options. For decades, practicians specialized in the management of BC have introduced the notion of “therapeutic de-escalation”, allowing, for instance, to avoid chemotherapeutic treatments, which are unnecessary for some patients. How to select patients eligible for therapeutic de-escalation? Research stays focused on biomarkers discovery: these biomarkers or tests should ideally predict the risk of recurrence. Gene-expression signature (GES, reviewed here) can help practicians to better select patients with low recurrence risk for whom chemotherapy can be avoided. GES can now be used to decipher the heterogeneity of BC and is used for decisions making in early breast cancer (notably in ER^+^/HER2^−^ early BC [4], chemotherapy being only indicated for high risk/score). It is now accepted that the patients with HR^+^/HER2^−^ early BC and having a low genomic risk score can safely skip neoadjuvant or adjuvant chemotherapy. This test is expected to spare up to 70,000 patients a year only in the United States [97]. The use of first-generation GES in patients with 1–3 positive lymph nodes is, however, still controversial, as only few prospective trials have so far been reported [4]. Finally, GES has still not yet proven beneficial in clinical trials for advanced BC but may be used in the context of prospective molecular triage programs to select patients for therapeutic trials [98].

Similarly to GES, some biomarkers can also help predict the risk of recurrence. Urokinase-type plasminogen activator (uPA) and its inhibitor, plasminogen activator inhibitor type 1 (PAI-1) have a high prognostic impact [99]. A high level of the two biomarkers is associated with poor PFS and poor OS [23,100]. Latest ESMO guidelines recommend that “expression of uPA-PAI1 of multigene panels may be used in conjunction with all clinico-pathological factors to guide systemic treatment decisions” in pre-selected patients.

When no benefits are expected from chemotherapy regimens, new solutions have to be used to improve patients’ outcomes. The aim of this review was also to highlight three of the most helpful and/or promising recent molecules in BC treatments to date (one for each main BC subtype: one for HER2^+^ patients (neratinib), one for TNBC patients (atezolizumab), and one for HR^+^/HER2^−^ patients (palbociclib).

### 5.1. For HER2^+^ Patients

Neratinib is the only treatment allowing significant decrease in relapses after one year trastuzumab treatment. Indeed, two years of adjuvant trastuzumab is not more effective than one year of treatment for patients with HER2^+^ early BC [101]. Neratinib obtained authorization in many countries (such as USA and France, since 2018) for patients with early HR^+^/HER2^+^ BC after trastuzumab treatment (and up to one year after the end of the treatment). In the last recommendations from the European Society for Medical Oncology (ESMO), “neratinib may be considered in selected high-risk patients, not previously treated with dual blockade, and with appropriate diarrhea prophylaxis and management” [98]. Other TKIs are not necessarily as efficient: it has been shown that sunitinib (which inhibits VEGF receptors), either alone or in combination with chemotherapy, had no clinical benefit for patients with advanced BC [102]. Lapatinib (which inhibits HER2 and EGFR) has been used since 2007 for metastatic HER2-positive BC [103] and shows efficiency on PFS and/or OS for patients with HER2^+^ BC in association with capecitabine [104], trastuzumab [105], or Letrozole [106]. Alfatinib, which inhibits HER1, HER2, and HER4, was stopped in early phase III due to unfavorable benefit–risk [107] (it is however used in non-small-cell lung cancer).

### 5.2. For TNBC Patients

The humanized monoclonal anti-PD-L1 antibody (atezolizumab) was demonstrated to be efficient on PFS in advanced or metastatic TNBC (results are not significant for OS). Positive effects were also found on partial or complete response. Atezolizumab’s efficiency is better when used in association with Nab-Paclitaxel. Difference in terms of PFS gain may appear very small, but patients with metastatic TNBC have a really poor prognosis. Last ESMO recommendations suggest that for tumors with mismatch repair deficiency, anti-PD-L1 can be used but with a weak level of evidence and grade of recommendation [98]. Atezolizumab is used on a case-by-case basis, when PD-L1 is expressed in more than 1% of immune cells. The first anti-PD-L1 used in cancer was ipilimumab, which proved important efficiency in melanoma. It increased overall response rate (ORR) by 11% in monotherapy [108], and by 61% when it was associated with another anti-PD-L1, nivolimumab [109]. The same association showed benefits in two-year OS in patients with advanced melanoma [108].

### 5.3. For HR^+^/HER2^−^ Patients

Palbociclib is an anti CDK4/6 inhibitor, which is able to increase up to 24 months the median PFS rate. Palbociclib efficiency is higher when it is used in association with fulvestrant. In Europe, Palbociclib is recommended for patients with advanced or metastatic HR^+^ and HER2^−^ BC, either in association with anti-aromatase or with letrozole or fulvestrant for patients having already received endocrine therapy. Latest ESMO guidelines recommend, for these patients: “addition of a CDK 4/6 inhibitor to an aromatase inhibitor (AI), in patients naıve or pre-exposed to endocrine therapy, is one of the preferred treatment options for pre- and peri-menopausal women and post-menopausal women” and also “addition of a CDK 4/6 inhibitor to fulvestrant, in patients previously exposed to endocrine therapy is one of the preferred treatment options, if a CDK 4/6 inhibitor was not previously used, for pre- and peri-menopausal women and post- menopausal women” [98]. Ribociclib and Abemaciclib are other CDK 4/6 inhibitors used in metastatic or locally advanced HR^+^/HER2^−^ BC treatment, in association with AI or fulvestrant. For such patients (post-menopausal), Ribociclib and Abemaciclib significantly improved median PFS and ORR in association with Letrozole [92,93].

## 6. Conclusions

Collaboration between fundamental and clinical research has allowed the discovery of new therapies, such as neratinib, atezolizumab, and palbociclib, which have proven their effectiveness for patients. Many treatments, molecules, and associations are still under investigation, and many others may show their effectiveness in the future. Other molecules, such as ribociclib, are of interest, but neratinib, palbociclib, and atezolizumab are currently still studied in adjuvant or neo-adjuvant settings, in association with chemotherapy or other new therapies (such as Talimogene Laherparepvec, which is an oncolytic virotherapy). Personalized medicine requires clinical, but also molecular, characterization of the tumors (with genomic tests for instance) that allows for substitution of chemotherapies with newer, more suitable drugs. This way, improvements have already been obtained for early BC: the next challenge will be to improve advanced BC therapy.

## Supplementary Materials

The following are available online at https://www.mdpi.com/2072-6694/12/9/2634/s1, Table S1: Lists of genes analyzed in each GES, Table S2: Data from phase II studies about neratinib, Table S3: Atezolizumab in TNBC, Table S4: Palbociclib in phase II and III studies.
