# Supplementary material for: Beyond Chemotherapies: Recent Strategies in Breast Cancer Treatment
Source: Cancers (Basel). 2020 Sep 16;12(9):2634. doi: 10.3390/cancers12092634 (PMC7565588; doi:10.3390/cancers12092634)
Supplement: Supplementary file 1 [file cancers-12-02634-s001.pdf]

Supplementary Materials: Beyond Chemotherapies: Recent Strategies in Breast Cancer Treatment

Arthur Foulon, Pierrick Theret, Lise Rodat-Despoix and Philippe Kischel

Table S1. Lists of genes analyzed in each Gene Expression Signature (GES).

OncotypeDX [1]	Mammaprint [2]	Prosigna PAM50 [3]	Endopredict [4]
beta-actin	*AL080059*	*FOXC1*	*AZGP1*
GAPDH	*Contig 63649RC*	*MIA*	*IL6ST*
GUS	*MMP9*	*KNTC2*	*RBBP8*
RPLPO	*DC13*	*CEP55*	*DHCR7*
TFRC	*EXT1*	*ANLN*	*MGP*
Ki67	*AL137718*	*MELK*	*STC2*
STK15	*HEC*	*GPR160*	*BIRC5*
Survivin	*ECT2*	*TMEM45B*	*UBE2C*
cyclin B1	*GMPS*	*ESR1*	*CALM2*
MYBL2	*Contig 32185RC*	*FOXA1*	*OAZ1*
GRB7	*Contig 35251RC*	*ERBB2*	*RPL37A*
HER2	*GNAZ*	*GRB7*	
ER	*SERF1A*	*FGFR4*	
PGR	*OXCT*	*BLVRA*	
BCL2	*ORC6L*	*BAG1*	
SCUBE2	*L2DTL*	*CDC20*	
MMP11	*PRC1*	*CCNE1*	
Stromelysin 3	*AF052162*	*ACTR3B*	
Cathepsin L2	*Contig 55725RC*	*MYC*	
GSTM1	*CENPA*	*SFRP1*	
CD68	*SM20*	*KRT14*	
BAG1	*MCM6*	*KRT5*	
	AKAP2	*MLPH*	
	Contig 56457RC	*CCNB1*	
	MP1	*CDC6*	
	Contig 40831RC	*TYMS*	
	Contig 51464RC	*UBE2T*	
	IGFBP5	*RRM2*	
	CCNE2	*MMP11*	
	ESM1	*CXXC5*	
	Contig 20217RC	*ORC6L*	
	LOC57110	*MDM2*	
	AP2B1	*KIF2C*	
	CFFM4	*PGR*	
	PECI	*MKI67*	
	TGFB3	*BCL2*	
	Contig 46223RC	*EGFR*	
	BBC3	*PHGDH*	
	CEGP1	*CDH3*	
	Contig 48328RC	*NAT1*	
	ALDH4 KIAA1442	*SLC39A6*	
	Contig 32125RC	*MAPT*	
	LOC51203	*UBE2C*	
	Contig 46218RC	*PTTG1*	
	Contig 38288RC	*EXO1*	
	AA555029RC	*CENPF*	
	FGF18	*CDCA1*	
	PK428	*MYBL2*	

OncotypeDX [1]	Mammaprint [2]	Prosigna PAM50 [3]	Endopredict [4]
	UCH37	*BIRC5*	
	KIAA1067		
	COL4A2		
	KIAA0175		
	RAB6B		
	Contig 28552RC		
	WISP1		
	RFC4		
	DKFZP564D0462		
	DCK		
	SLC2A3		
	Contig 24252RC		
	FLJ11190		
	NMU		
	Contig 63102RC		
	PECI		
	IGFBP5		
	Contig 55377RC		
	HSA250839		
	GSTM3		
	FLT1		

Table S2. Data from phase II studies about neratinib.

Author	Treatment	Number of Patients	Median PFS	CR %, (*n*) *	PR %, (*n*) *	Stable ≥ 24 Weeks *	Progression *
Blackwell et al. [5]	Neratinib + trastuzumab	28	15.9 weeks	7.1 (2)	21.4 (6)	7.1 (2)	21.4 (6)
Martin et al. [6]	Neratinib vs. lapatinib + capecitabine	117 vs. 116	4.5 months vs. 6.8 months (NS)	1.7 (2)	27.3 (32)	15.4 (18)	17.1 (20)
Awada et al. [7]	Neratinib + paclitaxel vs. trastuzumab + paclitaxel	242 vs. 237	12.9 months vs. 12.9 months (NS)	1.7 (4)	73.1 (117)	/	/
Chow et al. [8]	Neratinib + paclitaxel	99	57 weeks	7 (7)	66.7 (66)	9 (9)	6.1 (6)
Burstein et al. [9]	Neratinib	136	22.3 weeks ** 39.6 weeks ***	0.8 (1)	36.8 (50)	10.3 (14)	16.2 (22)
Freedman et al. [10]	Neratinib	40	1.9 months	0	7.5 (3)	/	
Park et al. [11]	Neratinib (neoadjuvant)	211	/	/	/	/	/
	Total neratinib	873		2.4% (16/662)	41.4% (274/662)	11.3% (43/380)	14.2% (54/380)

* neratinib group; ** with prior trastuzumab; *** without prior trastuzumab. PFS: progression free survival, CR: complete response, PR: partial response, NS: Not Significant. (*n*) = number of patients concerned.

Table S3. Atezolizumab in TNBC.

Author	Treatment	Number of Patients	Median PFS * (CI 95%)	Median OS * (CI 95%)	CR % (*n*)	PR % (*n*)	Progression	Median Follow-Up *
Schmid et al. [12]	Atezolizumab + nab-paclitaxel vs. Placebo + nab-paclitaxel	451 vs. 451	7.2 (5.6–7.5) vs. 5.5 (5.3–5.6)	21.6 (17.3-–3.4) vs. 17.6 (15.0–20.0)	7.1 (32)	48.8 (220)	15.3 (69)	12.9
Adams et al. [13]	Atezolizumab + nab-paclitaxel	33	5.5 (5.1–7.7)	14.7 (10.1–NE)	3 (1)	36.4 (12)	18.2 (6)	24.4
Emens et al. [14]	Atezolizumab	116	1.4 (1.3–1.6)	8.9 (7.0–12.6)	2.6 (3)	6.9 (8)	62.9 (73)	25.3

* Value in months. PFS: progression free survival, OS: overall survival, CR: complete response, PR: partial response, NE: Not Estimable, CI: confidence interval. (*n*) = number of patients concerned.

Table S4. Palbociclib in phase II and III studies.

Phase	Author	Treatment	Patients	Median PFS (95% CI) *	Median OS (95% CI) *	CR % (*n*)	PR % (*n*)	Progression % (*n*)	Stable Disease ≥24 Weeks	Median Follow-Up* (95% CI)
II	DeMichele et al. [15]	Palbociclib	33	3.8 (1.9–5.8)	/	0	6.1 (2)	39.4 (13)	/	UN
	Finn et al. *(PALOMA-1)* [16]	Palbociclib + letrozole vs.	84 vs.	20.2 (13.8–27.5) vs.	/	1.2 (1)	41.7 (35)	3.6 (3)	38.1 (32)	29.6 (27.9-36)

		letrozole	81	10.2 (5.7–12.6) (1)	/					
	Brufsky et al. [17]	Palbociclib + letrozole	126	4.5 (3.7–6.2)	21,1 (14,8–NE)	1,6 (2)	4 (5)	35.7 (45)	27.8 (35)	14,2
		Total	243			1.2 (3)	17.3 (42)	25.1 (61)	27.6 (67)	
III	Finn et al. *(PALOMA-2)* [18]	Palbociclib + letrozole vs.	444	24.8 (22.1–NE) vs.		/	/	/		23
		placebo + letrozole	222	14.5 (12.9–17.1)						
	Cristofanilli et al. *(PALOMA-3)* [19]	palbociclib + fluvestrant vs.	347 vs.	9.5 (9.2–11)		0	19 (66)	17 (58)		8.9
		placebo + fluvestrant	174	4.6 (3.5–5.6)						

* Value in months. PFS: progression free survival, OS: Overall survival, CR: complete response, PR: partial response, CI: confidence interval. (*n*) = number of patients concerned.

References

1. Paik, S.; Tang, G.; Shak, S.; Kim, C.; Baker, J.; Kim, W.; Cronin, M.; Baehner, F. L.; Watson, D.; Bryant, J.; et al. Gene expression and benefit of chemotherapy in women with node-negative, estrogen receptor-positive breast cancer. *J. Clin. Oncol.: Off. J. Am. Soc. Clin. Oncol.* **2006**, *24*, 3726–3734.
2. van 't Veer, L. J.; Dai, H.; van de Vijver, M. J.; He, Y. D.; Hart, A. A.; Mao, M.; Peterse, H. L.; van der Kooy, K.; Marton, M. J.; Witteveen, A. T.; et al. Gene expression profiling predicts clinical outcome of breast cancer. *Nature* **2002**, *415*, 530–536.
3. Parker, J. S.; Mullins, M.; Cheang, M. C.; Leung, S.; Voduc, D.; Vickery, T.; Davies, S.; Fauron, C.; He, X.; Hu, Z.; et al. Supervised risk predictor of breast cancer based on intrinsic subtypes. *J. Clin. Oncol.: Off. J. Am. Soc. Clin. Oncol.* **2009**, *27*, 1160–1167.
4. Filipits, M.; Rudas, M.; Jakesz, R.; Dubsky, P.; Fitzal, F.; Singer, C. F.; Dietze, O.; Greil, R.; Jelen, A.; Sevelda, P.; et al. A new molecular predictor of distant recurrence in ER-positive, HER2-negative breast cancer adds independent information to conventional clinical risk factors. *Clin. Cancer Res.: Off. J. Am. Assoc. Cancer Res.* **2011**, *17*, 6012–6020.
5. Blackwell, K. L.; Zaman, K.; Qin, S.; Tkaczuk, K. H. R.; Campone, M.; Hunt, D.; Bryce, R.; Goldstein, L. J. Neratinib in Combination With Trastuzumab for the Treatment of Patients With Advanced HER2-positive Breast Cancer: A Phase I/II Study. *Clin. Breast Cancer* **2019**, *19*, 97–104.
6. Martin, M.; Bonneterre, J.; Geyer, C. E., Jr.; Ito, Y.; Ro, J.; Lang, I.; Kim, S.-B.; Germa, C.; Vermette, J.; Wang, K.; et al. A phase two randomised trial of neratinib monotherapy versus lapatinib plus capecitabine combination therapy in patients with HER2+ advanced breast cancer. *Eur. J. Cancer* **2013**, *49*, 3763–3772.
7. Awada, A.; Colomer, R.; Inoue, K.; Bondarenko, I.; Badwe, R. A.; Demetriou, G.; Lee, S. C.; Mehta, A. O.; Kim, S. B.; Bachelot, T.; et al. Neratinib Plus Paclitaxel vs Trastuzumab Plus Paclitaxel in Previously Untreated Metastatic ERBB2-Positive Breast Cancer: The NEfERT-T Randomized Clinical Trial. *Jama Oncol.* **2016**, *2*, 1557–1564.
8. Chow, L. W.; Xu, B.; Gupta, S.; Freyman, A.; Zhao, Y.; Abbas, R.; Vo Van, M. L.; Bondarenko, I. Combination neratinib (HKI-272) and paclitaxel therapy in patients with HER2-positive metastatic breast cancer. *Br. J. Cancer* **2013**, *108*, 1985–1993.
9. Burstein, H. J.; Sun, Y.; Dirix, L. Y.; Jiang, Z.; Paridaens, R.; Tan, A. R.; Awada, A.; Ranade, A.; Jiao, S.; Schwartz, G.; et al. Neratinib, an Irreversible ErbB Receptor Tyrosine Kinase Inhibitor, in Patients With Advanced ErbB2-Positive Breast Cancer. *J. Clin. Oncol.* **2010**, *28*, 1301–1307.
10. Freedman, R. A.; Gelman, R. S.; Wefel, J. S.; Melisko, M. E.; Hess, K. R.; Connolly, R. M.; Van Poznak, C. H.; Niravath, P. A.; Puhalla, S. L.; Ibrahim, N.; et al. Translational Breast Cancer Research Consortium (TBCRC) 022: A Phase II Trial of Neratinib for Patients With Human Epidermal Growth Factor Receptor 2-Positive Breast Cancer and Brain Metastases. *J. Clin. Oncol.: Off. J. Am. Soc. Clin. Oncol.* **2016**, *34*, 945–952.
11. Park, J. W.; Liu, M. C.; Yee, D.; Yau, C.; van 't Veer, L. J.; Symmans, W. F.; Paoloni, M.; Perlmutter, J.; Hylton, N. M.; Hogarth, M.; et al. Adaptive Randomization of Neratinib in Early Breast Cancer. *New Engl. J. Med.* **2016**, *375*, 11–22.
12. Schmid, P.; Adams, S.; Rugo, H. S.; Schneeweiss, A.; Barrios, C. H.; Iwata, H.; Dieras, V.; Hegg, R.; Im, S. A.; Shaw Wright, G.; et al. Atezolizumab and Nab-Paclitaxel in Advanced Triple-Negative Breast Cancer. *New Engl. J. Med.* **2018**, *379*, 2108–2121.
13. Adams, S.; Diamond, J. R.; Hamilton, E.; Pohlmann, P. R.; Tolaney, S. M.; Chang, C. W.; Zhang, W.; Iizuka, K.; Foster, P. G.; Molinero, L.; et al. Atezolizumab Plus nab-Paclitaxel in the Treatment of Metastatic Triple-Negative Breast Cancer With 2-Year Survival Follow-up: A Phase 1b Clinical Trial. *Jama Oncol.* **2019**, *5*, 334–342.
14. Emens, L. A.; Cruz, C.; Eder, J. P.; Braiteh, F.; Chung, C.; Tolaney, S. M.; Kuter, I.; Nanda, R.; Cassier, P. A.; Delord, J. P.; et al. Long-term Clinical Outcomes and Biomarker Analyses of Atezolizumab Therapy for Patients With Metastatic Triple-Negative Breast Cancer: A Phase 1 Study. *Jama Oncol.* **2019**, *5*, 74–82.
15. DeMichele, A.; Clark, A. S.; Tan, K. S.; Heitjan, D. F.; Gramlich, K.; Gallagher, M.; Lal, P.; Feldman, M.; Zhang, P.; Colameco, C.; et al. CDK 4/6 inhibitor palbociclib (PD0332991) in Rb+ advanced breast cancer: Phase II activity, safety, and predictive biomarker assessment. *Clin. Cancer Res.: Off. J. Am. Assoc. Cancer Res.* **2015**, *21*, 995–1001.
16. Finn, R. S.; Crown, J. P.; Lang, I.; Boer, K.; Bondarenko, I. M.; Kulyk, S. O.; Ettl, J.; Patel, R.; Pinter, T.; Schmidt, M.; et al. The cyclin-dependent kinase 4/6 inhibitor palbociclib in combination with letrozole

versus letrozole alone as first-line treatment of oestrogen receptor-positive, HER2-negative, advanced breast cancer (PALOMA-1/TRIO-18): A randomised phase 2 study. *Lancet Oncol.* **2015**, *16*, 25–35.
17. Brufsky, A.; Mitra, D.; Davis, K. L.; Nagar, S. P.; McRoy, L.; Cotter, M. J.; Stearns, V. Treatment Patterns and Outcomes Associated With Palbociclib Plus Letrozole for Postmenopausal Women With HR(+)/HER2(−) Advanced Breast Cancer Enrolled in an Expanded Access Program. *Clin. Breast Cancer* **2019**, *19*, 317–325.
18. Finn, R. S.; Martin, M.; Rugo, H. S.; Jones, S.; Im, S.-A.; Gelmon, K.; Harbeck, N.; Lipatov, O. N.; Walshe, J. M.; Moulder, S.; et al. Palbociclib and Letrozole in Advanced Breast Cancer. *New Engl. J. Med.* **2016**, *375*, 1925–1936.
19. Cristofanilli, M.; Turner, N. C.; Bondarenko, I.; Ro, J.; Im, S.-A.; Masuda, N.; Colleoni, M.; DeMichele, A.; Loi, S.; Verma, S.; et al. Fulvestrant plus palbociclib versus fulvestrant plus placebo for treatment of hormone-receptor-positive, HER2-negative metastatic breast cancer that progressed on previous endocrine therapy (PALOMA-3): Final analysis of the multicentre, double-blind, phase 3 randomised controlled trial. *Lancet Oncol.* **2016**, *17*, 425–439.

© 2020 by the authors. Licensee MDPI, Basel, Switzerland. This article is an open access article distributed under the terms and conditions of the Creative Commons Attribution (CC BY) license (http://creativecommons.org/licenses/by/4.0/).
